# Genome-Wide Analysis of the *PR1* Gene Family in *Pinus massoniana* Under *Bursaphelenchus xylophilus* Stress

**DOI:** 10.3390/plants15091325

**Published:** 2026-04-26

**Authors:** Haiyu Zhou, Qingyang Chen, Shan Hu, Zhichun Zhou, Kai Gao, Bin Liu, Qinghua Liu

**Affiliations:** 1 Research Institute of Subtropical Forestry, Chinese Academy of Forestry, Hangzhou 311400, China; 15029702710@163.com (H.Z.); 13345012802@163.com (Q.C.); lkyhushan@163.com (S.H.); zczhou_risf@163.com (Z.Z.); gaokai@caf.ac.cn (K.G.); 2 College of Landscape Architecture, Nanjing Forestry University, Nanjing 210037, China; 3 National Key Laboratory of Forest Genetics and Tree Breeding, Chinese Academy of Forestry, Beijing 100091, China

**Keywords:** *Pinus massoniana*, pathogenesis-related protein 1, *Bursaphelenchus xylophilus*, gene cluster, expression analysis

## Abstract

Pathogenesis-related protein 1 (PR1) plays important roles in plant responses to both biotic and abiotic stresses; however, its role in mediating defense against pine wood nematode in *Pinus massoniana* remains unclear. In this study, a total of 63 PR1 family members were identified in *P. massoniana* using bioinformatics approaches and were named *PmPR1-1* to *PmPR1-63* based on their phylogenetic relationships. Phylogenetic analysis showed that these members were distributed among four of the six subfamilies. Most of the encoded proteins were hydrophilic, with lengths ranging from 131 to 406 amino acids. Their promoter regions contained multiple cis-acting elements associated with phytohormone signaling and stress responses, and some members formed gene clusters on chromosomes 2, 5, and 9. qRT-PCR (quantitative reverse transcription polymerase chain reaction) analysis showed that the clustered genes *PmPR1-46*, *PmPR1-55*, *PmPR1-56*, and *PmPR1-61* were significantly upregulated in the early stage of pine wood nematode inoculation in both resistant and susceptible *P. massoniana* plants, with higher expression levels in resistant plants. Transient overexpression of *PmPR1-61* increased SOD and PPO activities as well as proline content while decreasing CAT activity. These results suggest that the *PmPR1* family may be involved in the defense response of *P. massoniana* against pine wood nematode. Among them, *PmPR1-55*, *PmPR1-56*, and *PmPR1-61* represent candidate resistance genes worthy of further investigation and provide valuable gene resources for elucidating resistance mechanisms and supporting molecular breeding in *P. massoniana*.

## 1. Introduction

*Pinus massoniana* Lamb. is an evergreen arbor species belonging to the genus *Pinus* in the family Pinaceae. Owing to its strong adaptability, drought tolerance, and tolerance to poor soils, it is a pioneer tree species widely used for afforestation of barren hills in southern China. It is extensively utilized in pulp and paper production, construction, and resin tapping, and plays an essential role in the establishment of fast-growing timber forests and oleoresin-producing forest bases in China [1]. However, the pine industry is currently facing a serious threat from pine wilt disease caused by *Bursaphelenchus xylophilus*. According to an announcement issued by the National Forestry and Grassland Administration in 2025, this disease has spread to 18 provinces (autonomous regions and municipalities) in China, causing severe economic and ecological losses. Although chemical control and the removal of infected wood have, to some extent, slowed the spread of the disease, breeding resistant varieties is still regarded as the most economical and sustainable strategy for the effective control of pine wilt disease in *P. massoniana* in the long term [2]. Therefore, identifying key genes involved in resistance to pine wilt disease and elucidating their molecular mechanisms are of great significance for resistance breeding in *P. massoniana*.

Pathogenesis-related proteins (PRs), as important defensive proteins in plants, are widely distributed in higher plants. At present, 17 PR families have been identified, and different PR family members play distinct yet coordinated roles in plant defense responses [3,4]. For example, PR-2 exhibits β-1,3-glucanase activity, PR-3 exhibits chitinase activity, PR-5 exerts dual defensive functions by forming transmembrane pores or interfering with fungal signaling pathways, PR-9 exhibits peroxidase activity, PR-10 functions as a defensive protein in plant responses to adverse environmental stresses, and PR-12 to PR-14 confer broad-spectrum antimicrobial activity by disrupting pathogen membrane structures [5,6,7]. These findings indicate that PR family members participate in plant immune responses through multiple mechanisms, including degradation of pathogen cell walls, disruption of membrane structures, production of antimicrobial metabolites, and activation of systemic defense signaling. Among them, the PR1 family has attracted considerable attention because of its high sequence conservation and broad inducible expression characteristics. PR1 proteins usually contain a typical CAP domain, which is considered to be closely associated with their antimicrobial activity [8], suggesting that PR1 plays an important role in plant defense responses.

A large number of studies have demonstrated the functional diversity of *PR1* (*Pathogenesis-Related protein 1*) genes in plant defense responses. In terms of disease resistance, overexpression of *PR1* genes can significantly enhance plant resistance to various pathogens, including fungi, bacteria, and viruses [8]. For example, recombinant maize PR1 protein exhibits significant antifungal activity against *Fusarium oxysporum* [9]; in tomato (*Solanum lycopersicum*), *SlPR1* enhances immune responses by blocking the translocation of the effector protein FolSvp2 [10]; and in watermelon, overexpression of *ClPR1* significantly improves the resistance of susceptible cultivars to zucchini yellow mosaic virus (ZYMV) [11]. In addition, *PR1* genes are also involved in responses to abiotic stresses such as high salinity, drought, and low temperature [12]. For instance, in upland cotton (*Gossypium hirsutum*), *PR1* expression is upregulated under both fungal infection and drought stress [13], whereas *AsPR1-4* expression is significantly downregulated under salt, low-temperature, heavy metal, and drought stresses [8]. These results indicate that *PR1* genes play multiple roles in plant adaptation to complex environments.

Although the functions and regulatory mechanisms of *PR1* genes have been relatively well studied in many herbaceous plants, such as pepper, tomato, rice, and soybean, as well as in woody plants such as poplar and citrus [14,15,16,17,18,19], research on these genes in coniferous species remains very limited. As an important coniferous timber species in China, *P. massoniana* possesses a large and highly complex genome, which has to some extent hindered the systematic identification and functional characterization of related genes.

Based on this, the present study systematically identified members of the *PmPR1* gene family using the whole-genome data of *P. massoniana* and analyzed their physicochemical properties, phylogenetic relationships, chromosomal distribution, gene clusters, and conserved motifs through bioinformatics approaches. Furthermore, qRT-PCR was used to examine the expression patterns of these genes in resistant and susceptible plants at different time points after infection by pine wood nematode, and transient overexpression assays were performed to preliminarily verify the defensive function of the candidate gene *PmPR1-61*. This study aims to provide a theoretical basis for further revealing the roles of *P. massoniana* PR1 proteins in defense against pine wilt disease.

## 2. Results

### 2.1. Identification and Physicochemical Property Analysis of the PmPR1 Gene Family

In this study, PR1 family member sequences from *Arabidopsis thaliana*, *Populus trichocarpa*, *Oryza sativa*, and *Ginkgo biloba* were used as query sequences to screen the *P. massoniana* genome [19,20]. The candidate sequences were then further verified for the presence of the CAP domain (PF00188), and sequences lacking the CAP domain or containing incomplete domains were removed. Ultimately, 63 *PmPR1* family members were identified and named according to their phylogenetic relationships (Table 1). The number of family members was significantly higher than that in *A. thaliana*, *O. sativa*, and other species.

Analysis of the physicochemical properties of the 63 *PmPR1* genes revealed that the encoded proteins ranged from 131 to 406 amino acids in length. Their molecular weights varied from 14,284.02 to 45,797.16 Da, with PmPR1-59 encoding the smallest protein and PmPR1-45 the largest. The theoretical isoelectric points (pI) ranged from 4.28 to 9.46, with basic proteins accounting for approximately 63% of the total. The instability index values ranged from 28.53 to 55.59, and 38 proteins were predicted to be unstable, representing about 60% of the family members. The aliphatic index ranged from 55.35 to 93.42, with PmPR1-25 showing the lowest value and PmPR1-63 the highest. GRAVY analysis showed that, except for PmPR1-63, all other members had GRAVY values below 0 (ranging from −0.716 to −0.08), indicating that nearly all PmPR1 proteins are hydrophilic. In addition, approximately 83% (52 proteins) of the PmPR1 proteins were predicted to contain signal peptides. TMHMM-2.0 analysis further showed that, except for PmPR1-45, none of the PmPR1 proteins contained transmembrane helices. Collectively, these results suggest that PmPR1 proteins are secreted soluble proteins rather than membrane-bound proteins. Subcellular localization prediction indicated that most PmPR1 proteins were localized to the chloroplast and extracellular space, with approximately 40% predicted to localize to the extracellular space, 41% to the chloroplast, and 6.3% to the vacuole, whereas only a few were predicted to localize to the nucleus or cytoplasm.

### 2.2. Phylogenetic Analysis of the PR1 Gene Family

**Figure 1:**
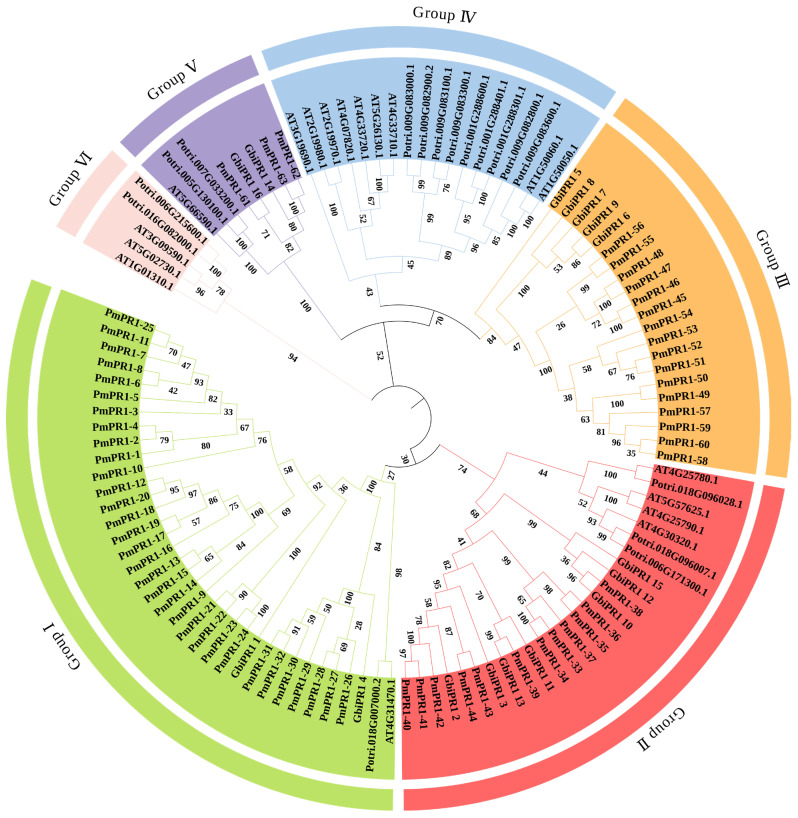
Phylogenetic tree of the *PR1* gene family. Note: At: Arabidopsis thaliana; Gbi: Ginkgo biloba; Pm: Pinus massoniana; Potri: Populus trichocarpa. Branches in different colors represent distinct subfamilies. Green: Group I; Red: Group II; Orange: Group III; Blue: Group IV; Purple: Group V; Pink: Group VI. Bootstrap values (based on 1000 replicates) are shown at the nodes.

To further clarify the evolutionary relationship of the *PmPR1s*, this study constructed a phylogenetic tree using MEGA12 software (Version 12.0.11)with 63 PR1 proteins identified from *P*. *massoniana*, 18 PR1 proteins from *A*. *thaliana*, 16 protein sequences from *G*. *biloba*, and 17 protein sequences from *P*. *trichocarpa* (Figure 1). Based on phylogenetic relationship analysis, all PR1 proteins were clustered into six groups, designated Group I to Group VI. Among them, Group I, Group II, Group III, and Group V contained members from *P*. *massoniana*, whereas Group IV and Group VI were composed exclusively of PR1 proteins from non-*P*. *massoniana* species and did not include any PmPR1 members. The number of PmPR1 family members varied considerably across these groups. Group I contained 32 PmPR1 proteins (PmPR1-1 to PmPR1-32); Group II was composed of 12 PmPR1 proteins (PmPR1-33 to PmPR1-44) and clustered with *AT4G25780.1*, indicating a close relationship. Previous studies have shown that *AT4G25780.1* is closely related to the basic chitinase HCHIB, which has antifungal activity and is associated with the JA/ET (Jasmonic acid, Ethylene) mediated signaling pathway. Therefore, it is speculated that the PmPR1 members clustered with it may have similar functions. Group III consisted of 16 PmPR1 proteins (PmPR1-45 to PmPR1-60); Groups IV and VI were composed exclusively of PR1 proteins from *A*. *thaliana* and *P*. *trichocarpa*; Group V included 3 PmPR1 proteins (PmPR1-61 to PmPR1-63), which clustered with *AT5G66590.1.* Previous studies have shown that *AT5G66590.1* interacts with PR4, which is highly related to the resistance of grape to downy mildew and is associated with the JA/ET signaling pathway [21]. Based on this, it is speculated that PmPR1-61 to PmPR1-63 may have certain functional associations in defense responses, although this hypothesis still requires further verification.

### 2.3. Analysis of the Gene Structure, Conserved Domains and Conserved Motifs of the PmPR1 Gene Family

**Figure 2:**
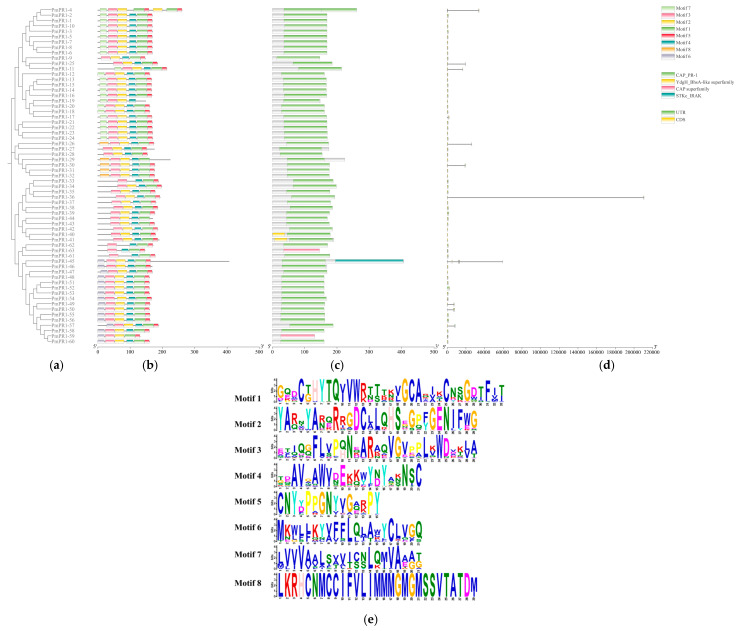
Gene structure and protein conserved motif distribution of *PmPR1*. Note: (**a**): Phylogenetic tree; (**b**): Conserved motifs of the protein encoded by the *PmPR1* gene; (**c**): Conserved domain architecture of PmPR1; (**d**): Gene structure of *PmPR1*; (**e**): Sequence logos of the eight motifs shown in panel (b).

Conserved motif analysis of the PmPR1 proteins was conducted using MEME (Version 5.5.9, https://meme-suite.org/meme/tools/meme, accessed on 3 April 2026) and Tbtools (Version 2.466), as shown in Figure 2a. Eight conserved motifs were identified in the PmPR1 proteins, among which five motifs (motif 1, motif 2, motif 3, motif 4, and motif 5) were consistently present in most family members, indicating high conservation. Additionally, motif 6 was mainly found in PmPR1-45 to PmPR1-60, motif 7 was mainly present in PmPR1-1 to PmPR1-23, and motif 8 was mainly observed in PmPR1-29 to PmPR1-32. The arrangement of these conserved motifs in the 63 PmPR1 protein members highlights the significant conservation within the *PmPR1* gene family. The results of the conserved domain analysis are shown in Figure 2b. All 63 PmPR1 proteins contain the CAP_PR-1 domain, which reinforces the conclusion that all members are part of the CAP superfamily.

The gene structures of the *PmPR1s* were analyzed using bioinformatics tools, and the results are shown in Figure 2c. Most of the genes are single-exon genes, with only 13 genes belonging to the multi-exon structure type. Among them, *PmPR1-1*, *PmPR1-19*, *PmPR1-29*, *PmPR1-32*, *PmPR1-40*, *PmPR1-41*, *PmPR1-42*, and *PmPR1-47*, these 8 members lack untranslated regions (UTRs). UTRs play important roles in the regulation of mRNA stability, transport, and translation in eukaryotes [22]. Therefore, the absence of UTR annotations in these genes may be related to limitations in the current annotation results, and their potential biological significance still requires further investigation.

### 2.4. Prediction of Cis-Acting Elements in the Promoter Regions of the PmPR1 Gene Family

**Figure 3:**
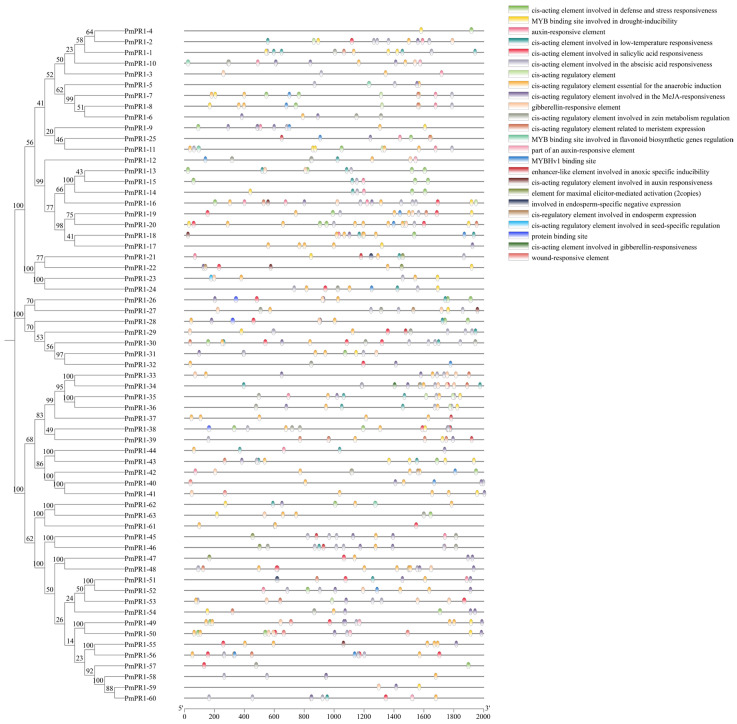
Analysis of cis-acting elements in the *PmPR1* gene family.Note: The 2-kb sequences upstream of the start codon were analyzed. Different colors represent different types of cis-acting elements.

To further investigate the potential biological processes and signaling pathways involved in the *PmPR1s*, this study extracted the 2000 bp upstream promoter sequences of the *PmPR1s* to analyze their cis-element characteristics. The results indicated that the promoter regions of the *PmPR1s* were enriched with a series of elements related to biotic and abiotic stress responses (defense and stress response, anaerobic induction, hypoxia-specific induction, drought induction, cold response, mechanical injury response), plant hormone interactions, and elements associated with plant growth and development (Figure 3).

Firstly, in terms of stress response, a total of 241 elements were identified to be involved in biotic and abiotic stress responses. Among them, elements related to defense and stress response were the most widely distributed, present in the promoters of 27 genes including *PmPR1-4*, *PmPR1-7*, *PmPR1-8*, and *PmPR1-9*, and these genes were evenly distributed across the four subfamilies of Group I, Group II, Group III, and Group IV, indicating that this function is relatively conserved in evolution. Additionally, specific elements such as mechanical injury response elements were mainly concentrated in the promoters of genes *PmPR1-40*, *PmPR1-41*, *PmPR1-49*, and *PmPR1-50* in the Group II and Group III subfamilies. Secondly, the largest number of elements were related to hormone responses, totaling 322, including elements for salicylic acid (SA), methyl jasmonate (MeJA), gibberellin (GA), abscisic acid (ABA), and auxin response. Notably, the presence of SA and MeJA response elements further confirmed the potential role of *PmPR1s* in plant stress resistance. For instance, Salicylic acid response elements are present in the promoters of 24 genes, including *PmPR1-2*, *PmPR1-16*, and *PmPR1-22*, mainly concentrated in the Group I and Group III subfamilies. Additionally, 36 elements related to plant growth and development were identified, such as auxin response elements, zein metabolism regulatory elements, seed-specific regulatory elements, and endosperm expression elements. The presence of these elements suggests that the *PmPR1s*, in addition to responding to stress, may also be involved in the growth and development of *P. massoniana*, performing diverse regulatory functions.

### 2.5. Mining of PmPR1s’ Gene Cluster in P. massoniana

**Figure 4:**
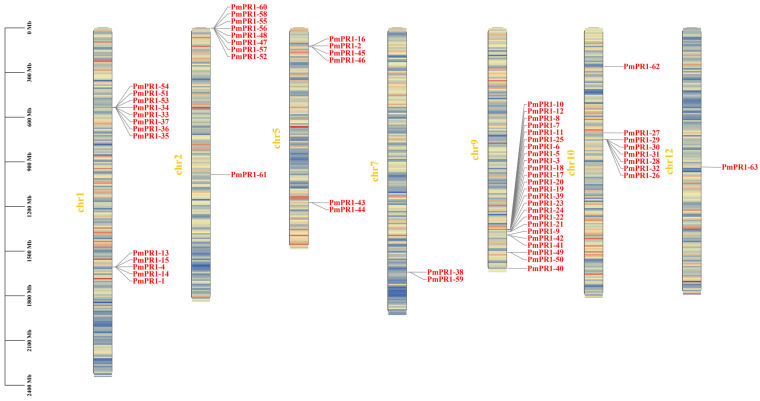
Chromosomal localization of the *PmPR1* gene family.Note: Red regions indicate high gene density, and blue regions indicate low gene density.

PR1, as an important component of the plant defense system, participates in the co-evolution of plants and pathogens and can form gene clusters through evolutionary events such as tandem and segmental duplications. Members of gene clusters can not only respond rapidly to pathogen infection through coordinated expression but also enhance plant disease resistance through functional differentiation. For instance, during the early and middle stages of *Fusarium oxysporum* infection in *Aleurites montana*, the *Aleurites montana PGK3-TPS10-CYP726* gene cluster was continuously highly expressed in the roots [23]; in soybeans, different members of the *RGC2* family have distinct functions and show different expression-related phenotypes [24]. Based on the genome annotation information of *P. massoniana*, the chromosomal localization of *PmPR1s* and the gene clusters they belong to were explored. The results are shown in Figure 4. *PmPR1s* are unevenly distributed on seven chromosomes of the *P. massoniana* genome, namely chromosomes 1, 2, 5, 7, 9, 10, and 12. Among them, chromosome 9 has the largest number of genes, with 24 genes, followed by chromosome 1 with 13 genes, and chromosome 12 has the fewest genes, with only the *PmPR1-63* located on it.

The *PmPR1s* are located on multiple chromosomes, including chromosome 1, chromosome 2, chromosome 7 and chromosome 9. A large number of *PmPR1s* are arranged in series, forming gene clusters. Among them, the *PmPR1s* located on chromosome 1 and belonging to the Group I subfamily form two clusters: the cluster composed of *PmPR1-4*, *PmPR1-13*, *PmPR1-14* and *PmPR1-15* has a total span of 72.8 kb, and the cluster composed of *PmPR1-51* and *PmPR1-54* has a total span of 63 kb. On chromosome 2, the *PmPR1s* belonging to the Group III subfamily also form two clusters: the cluster composed of *PmPR1-47*, *PmPR1-48* and *PmPR1-52* has a span of 291.7 kb, while the cluster composed of *PmPR1-58* and *PmPR1-60* has a span of 48.1 kb. The *PmPR1-10* and *PmPR1-12* gene cluster is located on chromosome 9, with a total span of 41.6 kb, and all members belong to the Group I subfamily. The *PmPR1-38* and *PmPR1-59* gene cluster is located on chromosome 7, with a total span of 327.8 kb. *PmPR1-38* belongs to the Group II subfamily, and *PmPR1-59* belongs to the Group III subfamily. This indicates that after forming gene clusters, *PR1* has undergone functional differentiation and participates in different disease resistance processes.

**Figure 5:**
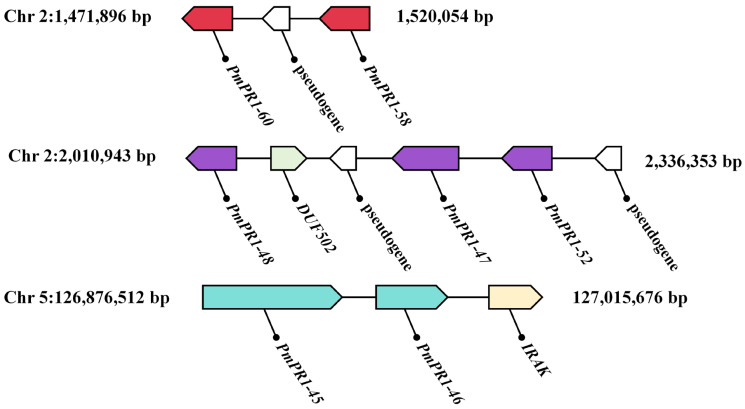
Schematic of the *PmPR1s’* gene cluster. Note: Boxes in different colors represent different gene clusters: red (chromosome 2, 1.47–1.52 Mb), purple (chromosome 2, 2.01–2.34 Mb), and green (chromosome 5, 126.88–127.02 Mb). Genes within boxes of the same color belong to the same gene cluster. The direction of the arrow in each box indicates gene orientation, and the length of each box is proportional to gene length. Chromosomal coordinates are shown in bp.

Based on the transcriptome data of high-resistant and susceptible *P. massoniana* at different infection stages obtained in the previous study of our research group, further analysis of the expression patterns of *PmPR1s* and gene clusters in high-resistant and susceptible *P. massoniana* at different infection nodes revealed that the expression of *PmPR1-45*, *PmPR1-46*, *PmPR1*-*48*, *PmPR1*-*51*, *PmPR1*-*52*, *PmPR1*-*53*, *PmPR1*-*55*, *PmPR1*-*56*, *PmPR1*-*58*, *PmPR1*-*60*, and *PmPR1*-*61* was particularly significant in resistant and susceptible plants. Moreover, *PmPR1*-*45* and *PmPR1*-*46* were clustered on chromosome 5, while *PmPR1*-*58* and *PmPR1*-*60* were clustered on chromosome 2. Additionally, *PmPR1*-*47*, *PmPR1*-*48*, and *PmPR1*-*52* were also clustered on chromosome 2 (Figure 5), indicating that their induction and activation can lead to rapid cluster expression [25].

**Figure 6:**
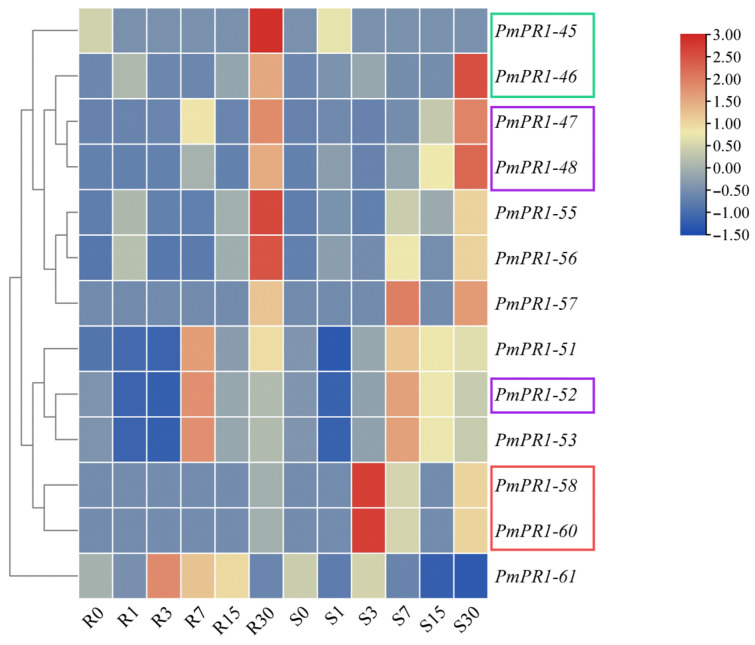
Transcript profiling of *PmPR1s* based on RNA-seq analysis. Note: Borders of the same color represent the same gene cluster. The red and purple borders indicate two different gene clusters on chromosome 2, whereas the green border indicates the gene cluster on chromosome 5.

As shown in Figure 6, different gene clusters exhibit distinct expression patterns in response to invasion in *P. massoniana*. The *PmPR1-58* and *PmPR1*-*60* gene clusters show the same trend of gene expression after infection by *B. xylophilus*, indicating their co-expression within the cluster; while the *PmPR1*-*45* and *PmPR1*-*46* gene clusters, as well as the *PmPR1*-*47*, *PmPR1*-*48* and *PmPR1*-*52* gene clusters, display specific expression in response to the invasion of *B. xylophilus*.

### 2.6. Expression Analysis of PmPR1s in Resistant and Susceptible P. massoniana at Different Periods After Nematode Inoculation

**Figure 7:**
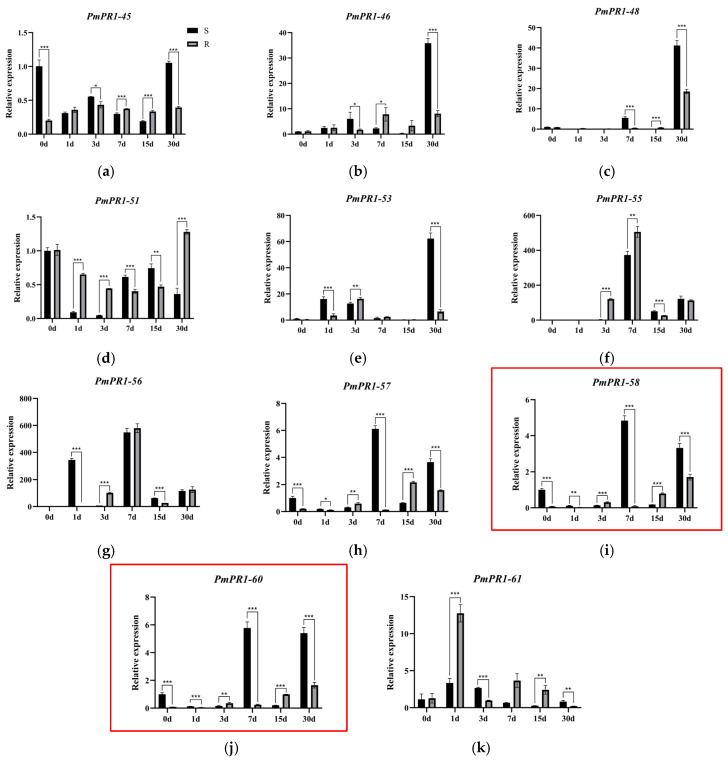
Quantitative real-time PCR (qRT-PCR) analysis of *PmPR1s’* expression. Note: The expression of *PmPR1* genes was validated by quantitative reverse transcription PCR (qRT-PCR). Panels (**a**–**k**) show the real-time fluorescence quantitative results for 11 different genes. The red box indicates genes that are located in the same gene cluster and show consistent expression trends. Relative expression levels were calculated using susceptible plants at 0 days post-inoculation (S0) as the control. Data are presented as mean ± standard deviation (SD) of three biological replicates. Asterisks indicate significant expression differences between resistant and susceptible plants (* *p* < 0.05; ** *p* < 0.01; *** *p* < 0.001; independent samples *t*-test).

To elucidate the expression patterns of *PmPR1s* in highly resistant (R) and susceptible (S) *P. massoniana* following inoculation with *B. xylophilus* over a 30-day period, key genes and gene clusters responsive to nematode infection in resistant and susceptible pines were selected for qRT-PCR analysis. Using 0, 1, 3, 7, 15, and 30 days post-inoculation as time points, Quantitative Real-Time PCR was performed on the 11 selected genes (Figure 7). The results revealed differential expression patterns among these genes, suggesting potential functional divergence. Using susceptible pines at 0 d post-inoculation (S0) as the control, distinct expression trends were observed for *PmPR1*-*45* and *PmPR1*-*46*. *PmPR1*-*45* exhibited an initial increase followed by stabilization in resistant plants after inoculation. In susceptible plants, its expression was significantly higher than in resistant plants at 0 d and 30 d post-inoculation, while no significant differences were observed at other time points. *PmPR1*-*46* reached peak expression in resistant plants at 7 d post-inoculation, whereas its induction in susceptible plants was delayed, with significantly higher expression than in resistant plants at 30 d post-inoculation. *PmPR1*-*58* and *PmPR1*-*60* showed similar expression trends, Synergistic response to pine wood nematode infection, with significant upregulation in susceptible clones at 7 d and 30 d post-inoculation. At 7 d post-inoculation, expression levels were approximately 5–6 times higher than at 0 d, representing a substantial increase. Specifically, at both time points, transcript levels in susceptible plants were significantly higher (approximately 2–5 times) than those in resistant plants. *PmPR1*-*48* showed no significant expression changes during early inoculation but was upregulated in both resistant and susceptible plants after 30 days, with significantly higher expression in susceptible plants. *PmPR1*-*51* displayed a downregulation-followed-by-upregulation pattern in both resistant and susceptible pines after inoculation, with higher expression in resistant plants. *PmPR1*-*55*, *PmPR1*-*56*, and *PmPR1*-*61* all exhibited an initial increase followed by a decrease in resistant plants, though with distinct response kinetics. At 1 d post-inoculation, *PmPR1*-*61* was rapidly induced in resistant plants, with expression levels approximately 3.7 times higher than in susceptible plants. *PmPR1*-*55* and *PmPR1*-*56* responded slightly later, showing rapid upregulation in resistant plants at 3 d and 7 d post-inoculation, respectively, with significantly higher expression than in susceptible plants. These findings indicate that the three *PR1* family genes (*PmPR1-55*, *PmPR1*-*56*, and *PmPR1*-*61*) may play critical roles in activating defense responses against *B. xylophilus* in *P. massoniana*. Given that *PmPR1*-*61* is rapidly induced during early resistance, potentially functioning upstream in defense signaling, it was selected for subsequent transient transformation experiments to validate its function.

### 2.7. Transient Transformation of PmPR1-61 in Callus of P. massoniana

**Figure 8:**
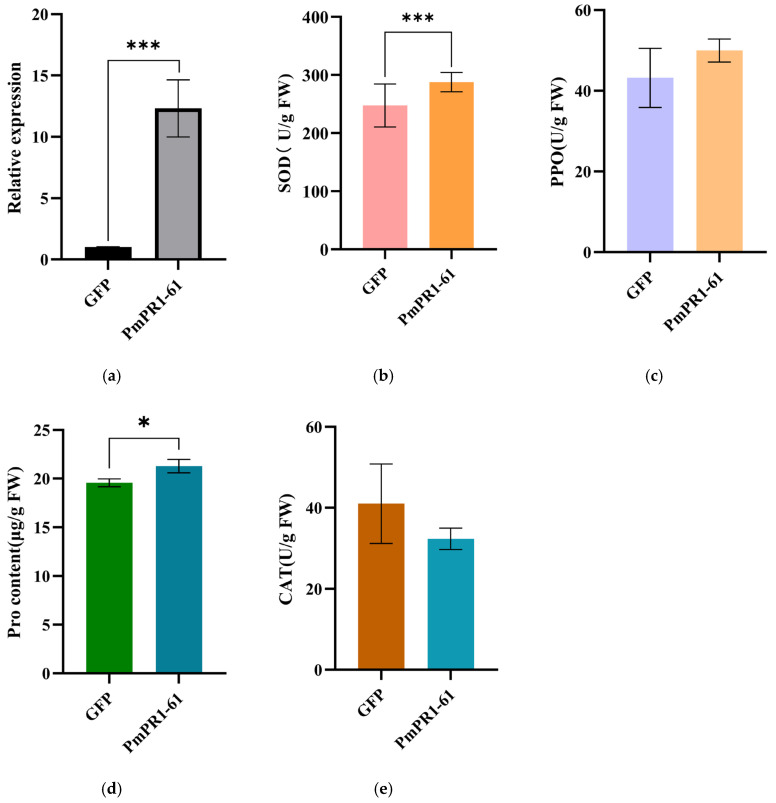
Physiological indicators of *P. massoniana* callus after transient overexpression of *PmPR1-61*. Note: (**a**): Expression analysis in *P. massoniana* callus after transient overexpression of *PmPR1-61*; (**b**): Superoxide dismutase (SOD) activity; (**c**): Polyphenol oxidase (PPO) activity; (**d**): Proline (PRO) content; (**e**): Catalase (CAT) activity. Data are presented as the mean ± standard deviation (SD) of three biological replicates. Asterisks indicate significant differences between the control (GFP) and transgenic (*PmPR1-61*) plants (* *p* < 0.05; *** *p* < 0.001; independent samples *t*-test).

To further explore the role of the *PmPR1-61* in activating the defense response of *P. massoniana* against *B. xylophilus*, an overexpression vector of this gene was constructed and transiently transformed into *P. massoniana* callus. The qRT-PCR results (Figure 8a) indicated that the relative expression level of the gene in the callus transiently transformed with pCAMBIA1300-*PmPR1-61*-EGFP was significantly higher than that in the control callus transiently transformed with the empty vector, approximately 12 times that of the control, suggesting the successful transient transformation of the *P. massoniana* callus.

This study further examined the activities of defense-related enzymes and the metabolite contents in overexpressing calli (Figure 8b–e). The results showed that, compared with the control, transient overexpression of *PmPR1-61* significantly increased superoxide dismutase (SOD) activity and proline (PRO) content (*p* < 0.05) while also enhancing polyphenol oxidase (PPO) activity; in contrast, catalase (CAT) activity decreased. SOD is a key enzyme responsible for scavenging superoxide anions, and its increased activity usually reflects the activation of the cellular antioxidant response. As one of the main enzymes involved in hydrogen peroxide (H_2_O_2_) decomposition, the reduced activity of CAT may indicate suppressed H_2_O_2_-scavenging capacity, thereby promoting H_2_O_2_ accumulation to activate downstream defense responses. Alternatively, it may reflect enzyme inactivation caused by oxidative damage, inhibition by post-translational modification, or an overall disruption of cellular redox homeostasis. Since H_2_O_2_ content was not directly measured in this study, these possibilities cannot currently be distinguished. The increases in PPO and PRO may indicate the establishment of an antimicrobial barrier and enhanced osmotic regulation capacity. In summary, transient overexpression of *PmPR1-61* altered the antioxidant enzyme system of the calli and promoted the accumulation of PPO and PRO. These changes may synergistically enhance the defense potential of *P. massoniana*, although the underlying mechanisms still require further verification through direct measurement of H_2_O_2_ levels and enzyme activity inhibition assays.

## 3. Discussion

PR-1 proteins are among the most abundantly synthesized proteins during plant immune responses and play a crucial role in SAR [26]. Although the PR1 family has been systematically identified in many angiosperms, related studies in Pinaceae species remain lacking. In this study, a total of 63 *PmPR1* genes were systematically identified for the first time in the conifer species *P*. *massoniana*. This number is markedly higher than that reported in *Populus deltoides* [20], *Pyrus betulaefolia Bunge* [27], and *Medicago sativa* [28], indicating that the PR1 family has undergone substantial expansion in *P. massoniana*. Combined with the chromosome localization results, *PmPR1s* were found to be tandemly distributed across multiple chromosomal regions, suggesting that tandem duplication may have been an important driving force for the expansion of this family in *P. massoniana*.

Most members of the *PmPR1* family lacked introns, a structural feature consistent with reports of *PR1* genes in poplar and other plant species [19,20]. Previous studies have shown that intronless or intron-poor genes are often more conducive to rapid transcriptional responses under stress conditions [25,29]. Therefore, the predominance of single-exon genes in the *PmPR1* family may provide a structural basis for the rapid activation of defense responses in *P*. *massoniana* during the early stage of pine wood nematode infection. However, such a structural advantage does not necessarily translate directly into stronger disease resistance, because whether a gene can be induced in a timely manner is also influenced by multiple factors, including the composition of cis-regulatory elements, chromatin accessibility, and the dynamic regulation of upstream transcription factors [5]. Therefore, the differential expression of *PmPR1* genes between resistant and susceptible plants is more likely the result of the combined effects of structural characteristics and regulatory networks.

Promoter analysis revealed that *PmPR1s* contain numerous cis-regulatory elements associated with SA, MeJA, ABA, and MYB/MYC, suggesting that the regulation of this family may be linked to multiple phytohormonal and stress-related signaling pathways [5,30]. It is generally accepted that the SA pathway is mainly associated with SAR-related resistance, whereas the JA/ET pathway is more commonly involved in responses to mechanical injury and insect stress [31]. Pine wood nematode infection is usually accompanied by multiple changes, including mechanical damage, tissue necrosis, and disruption of oxidative balance. Therefore, the coexistence of SA and MeJA responsive elements in *PmPR1* promoters suggests that members of this family may be coordinately regulated by multiple hormonal signals and participate in the crosstalk among different defense pathways [32]. Taken together, the differential expression of different *PmPR1* members may be associated with the defense demands at different stages of infection as well as changes in cellular physiological status during the infection process.

This study found that *PmPR1-61* was rapidly induced at 1 day after inoculation in highly resistant materials, whereas *PmPR1-55* and *PmPR1-56* were significantly upregulated at 3–7 days after inoculation. In contrast, *PmPR1-58* and *PmPR1*-*60* showed relatively high expression mainly at the late stage of infection in susceptible materials. The members that were rapidly upregulated at the early stage may be involved in the initiation of early defense responses, whereas those highly expressed at later stages in susceptible materials may be associated with stress responses or defense imbalance as the disease progresses [33,34,35]. These findings suggest that the *PmPR1* family may have undergone evident functional differentiation during resistance to nematode infection, with some members tending to function in early immune activation, while others may be involved in late defense maintenance or stress adaptation.

Studies on the relationship between PR1 proteins and reactive oxygen species (ROS) signaling have shown that ROS not only serve as important early defense signals during pathogen infection, but also act as major factors causing cellular oxidative damage [36,37]. In this study, transient overexpression of *PmPR1-61* led to increased SOD and PPO activities, elevated proline content, and reduced CAT activity. These results suggest that the role of *PmPR1-61* may extend beyond serving merely as a marker of defense responses, and that it may participate in the regulation of ROS homeostasis. In addition, the enhancement of PPO activity and the increase in proline accumulation after *PmPR1-61* overexpression indicate that its function is not limited to a single-antioxidant enzyme system. PPO is generally associated with the oxidation of phenolic compounds, tissue lignification, and the formation of physical barriers, whereas proline plays important roles in osmotic adjustment, membrane structure stabilization, and buffering against oxidative stress [38,39]. Therefore, *PmPR1*-*61* is more likely to enhance the defense capacity of *P*. *massoniana* through multiple mechanisms, including regulation of ROS signaling, reinforcement of cell wall defense, and maintenance of stress homeostasis. However, the decrease in CAT activity may also indicate an increase in local oxidative pressure, and thus the possibility that *PmPR1*-*61* enhances defense while simultaneously aggravating oxidative stress cannot yet be completely excluded. Further studies integrating in situ H_2_O_2_ staining, MDA determination, and related analyses are still needed to determine whether *PmPR1*-*61* enhances resistance mainly by regulating the dynamic changes of H_2_O_2_.

During the long-term co-evolution between plants and pathogens, the formation of disease-resistance gene clusters, such as *PR-1* and *R* genes, represents a key genomic adaptation strategy. Evolutionary events including tandem duplication and segmental duplication provide the genetic basis for the generation of duplicated genes. These duplicated genes can subsequently undergo functional divergence through neofunctionalization and subfunctionalization, thereby supplying abundant genetic resources for plant disease resistance [40,41,42]. Studies have shown that, in rice, different members within *NLR* gene clusters exhibit specific resistance to different races of Magnaporthe oryzae [43]. In *A*. *thaliana*, genes in the *RPW8/HR* cluster confer broad-spectrum disease resistance, whereas *RPP7* members provide race-specific resistance [44]. These findings indicate that functional differentiation within gene clusters is of great biological significance, manifesting either as broad-spectrum resistance or as specialized recognition and defense against specific pathogens.

Members of the *PR1* gene cluster in *P*. *massoniana* are distributed among different evolutionary branches. For example, *PmPR1*-*38* and *PmPR1*-*59* belong to Group II and Group III subfamilies, respectively, and may participate in defense against different pathogens in *P. massoniana*. Functional differentiation within gene clusters is reflected not only in nucleotide and amino acid sequences, but also in expression patterns. The key candidate disease-resistance gene *PmPR1*-*52*, together with *PmPR1*-*47* and *PmPR1*-*48*, forms a clustered distribution spanning 291.7 kb on chromosome 2. These genes display coordinated or specific expression patterns in different resistant materials and at different infection stages, suggesting that the gene cluster may regulate defense through coordinated action or functional divergence. Genes within the cluster may share similar combinations of promoter elements, such as SA, MeJA, and W-box-related binding sites, and are therefore likely to be co-regulated by the same class of defense-related transcription factors [5,20,30]. In addition, following pathogen infection, the chromatin in this genomic region may become more accessible, thereby facilitating the binding of relevant transcription factors and promoting the rapid induction of genes within the cluster [45,46].

In summary, members of the *PmPR1* gene family may exhibit both coordinated responses and functional differentiation during the resistance of *P*. *massoniana* to pine wood nematode, among which *PmPR1*-*55*, *PmPR1*-*56*, and *PmPR1*-*61* appear to be potential key resistance-related genes.

## 4. Materials and Methods

### 4.1. Materials

#### 4.1.1. Plant Materials

The experimental materials were collected from 8-year-old *P*. *massoniana* clonal trial forests in Linhai Nursery, Linhai City, Zhejiang Province. Three plants each of the highly resistant clone (1144-2) and the susceptible clone (9-5) were selected, and their current-year tender branches were inoculated with pine wood nematodes. The control group consisted of the susceptible masson pine clones inoculated 0 days ago. The pine wood nematodes used for inoculation were the highly pathogenic “Guangde 3B” and a mixed population of nematodes isolated from diseased masson pine trees in the wild. Each tender branch of *P*. *massoniana* was inoculated with 10,000 pine wood nematodes suspended in 200 μL. Three biological replicates of the highly resistant and susceptible *P*. *massoniana* clones inoculated with pine wood nematodes were collected at different time points (0, 1, 3, 7, 15, and 30 days), and immediately placed in liquid nitrogen and stored at −80 °C for subsequent experimental analysis.

#### 4.1.2. Data Sources

The genomic data of *P*. *massoniana* used in this study was obtained from the publicly available data of Guangxi Academy of Forestry Sciences (PRJNA1240911). The transcriptome data were the sequencing data from the previous work of our research group.

#### 4.1.3. Preparation of Culture Medium and Resuspension Solution

LB medium (1 L): 10 g NaCl + 10 g Tryptone + 5 g Yeast extract + 15 g Agar;

Co-culture medium (1 L): 4.48 g M519 + 30 g sucrose + 7.5 g agar, pH 5.8;

Resuspension solution: 10 mM/L MgCl_2_ + 10 mM/L MES + 200 μM/L AS.

### 4.2. Method

#### 4.2.1. Identification and Physicochemical Property Analysis of *PmPR1* Gene Family Members

The protein sequence of the pathogenesis-related protein *PR1* of *A*. *thaliana* was downloaded from the TAIR database (Version 10.0, https://www.arabidopsis.org/, accessed on 3 December 2025), the protein sequences of *PR1* in *O. sativa* and *P*. *trichocarpa* were downloaded from the NCBI official website (https://www.ncbi.nlm.nih.gov/gene/, accessed on 3 December 2025), and the PR1 protein sequence of *G*. *biloba* was obtained from published papers. Using the *PR1* protein sequences of *A*. *thaliana*, *O. sativa*, *P*. *trichocarpa* and the gymnosperm *G*. *biloba* as search objects, BLASTP analysis was performed on the *P*. *massoniana* genome data through Tbtools, with strict thresholds (E value ≤ 1× 10 ^−10^). To verify the existence of domains, the hidden Markov model file of the PR1 protein (PF00188) was downloaded from Pfam (Version 38.0, https://www.ebi.ac.uk/interpro/, accessed on 3 December 2025), and then HMM Search was conducted using the TBtools software (Version 2.466) to initially screen out candidate gene family members. The intersection of the results obtained by the above methods was taken, and the Batch CD-Search tool of NCBI (https://www.ncbi.nlm.nih.gov/Structure/bwrpsb/bwrpsb.cgi, accessed on 3 December 2025) was used to verify the domains of these candidate genes. Further, the physicochemical properties of the *PR1* family proteins of *P*. *massoniana* were analyzed using TBtools, including molecular weight, isoelectric point, instability index, hydrophobicity coefficient and total average hydrophilicity. Signal peptides were predicted using SignalP-6.0 (https://services.healthtech.dtu.dk/services/SignalP-6.0/, accessed on 6 April 2026), and transmembrane domains were predicted using TMHMM-1.0 (https://services.healthtech.dtu.dk/services/DeepTMHMM-1.0/, accessed on 6 April 2026). Finally, protein subcellular localization was predicted using the WoLF PSORT online platform with the plant mode selected (https://wolfpsort.hgc.jp/, accessed on 4 December 2025).

#### 4.2.2. Phylogenetic Analysis of *PmPR1* Gene Family

The *PR1* proteins of *P*. *massoniana*, *A*. *thaliana*, *G*. *biloba* and *P*. *tomentosa* were subjected to multiple sequence alignment using MEGA12 (Version 12.0.11) software. The evolutionary tree was constructed by the Maximum Likelihood Estimation (ML). The parameters were set as follows: the amino acid substitution model was JTT, Gaps/Missing Data was set to Use all sites, Tree Inference Options was set to NNI + Automatic initial tree, Number of Threads was set to 6, and bootstrap analysis was performed with 1000 replicates. Finally, the phylogenetic tree was visualized and annotated using the iTOL online tool (version 7.5.1, https://itol.embl.de/, accessed on 3 April 2026).

#### 4.2.3. Analysis of the Gene Structure Characteristics and Conserved Domains of the PmPR1s

The nucleic acid and protein sequences of the *PmPR1* family members were extracted from the genome annotation file of *P*. *massoniana*, including their complete genomic sequences, coding sequences (CDS), and corresponding protein sequences. On this basis, the gene structure diagrams were visualized using TBtools (Version 2.466), and the number and length of exons of each member were counted. To further explore the functional conservation, the conserved domain analysis was conducted using the Conserved domains software of NCBI (https://www.ncbi.nlm.nih.gov/Structure/bwrpsb/bwrpsb.cgi, accessed on 5 December 2025), and the conserved motif analysis was performed using MEME (Version 5.5.9, https://meme-suite.org/meme/tools/meme, accessed on 3 April 2026), with the parameters set to identify a maximum of 10 motifs and the distribution option set to zero or one occurrence per sequence (zoops), while all other parameters were kept at their default settings. Finally, the identified motifs were visualized using TBtools.

#### 4.2.4. Prediction of Cis-Acting Elements in the Promoter Regions of the *PmPR1* Gene Family

The 2000 bp sequence upstream of the start codon of the *PmPR1s* was extracted using the GFF3 sequence extraction tool in TBtools software (Version 2.466)and submitted to the PlantCare online website (https://bioinformatics.psb.ugent.be/webtools/plantcare/html/, accessed on 13 December 2025) to predict cis-acting elements. The cis-acting elements of the predicted promoter were visualized and analyzed using TBtools.

#### 4.2.5. Chromosome Mapping and Gene Cluster Mining of the *PmPR1* Gene Family

Based on the GFF annotation file of the *P*. *massoniana* genome data, the chromosomal location information of the *PmPR1* was obtained, and the visualization plot was made using the TBtools software. Adjacent *PmPR1* genes that are closely linked on the chromosome and belong to the same phylogenetic branch were defined as members of the same gene cluster. Gene location information was extracted from the genome GFF file and then visualized. The differentially expressed *PR1* genes in susceptible and resistant *P*. *massoniana* at different infection periods were screened out based on the transcriptome data, and the heat map of the differentially expressed genes in the *PmPR1* gene family of *P*. *massoniana* was drawn and analyzed.

#### 4.2.6. Analysis of the Expression Patterns of PmPR1s

To further investigate the functions of *PmPR1s* during infection by *B*. *xylophilus*, qRT-PCR analysis was conducted to verify the genes with significant differences in expression levels based on the gene cluster results and transcriptome data. Total RNA was extracted from the young branches of highly resistant and susceptible *P*. *massoniana* inoculated with *B*. *xylophilus* for different days using the Aidlab RNA extraction kit. The Aidlab reverse transcription kit was used to reverse transcribe 700 ng of total RNA into cDNA, which was then diluted three times for use. The thermal cycling program was as follows: 95 °C for 2 min; 95 °C for 15 s, 60 °C for 34 s, for 40 cycles. Specific primers were designed using the online software Primer3 plus (Version 3.2.0, https://www.primer3plus.com/, accessed on 24 November 2025) and synthesized. *EF2* was used as the internal reference gene, and the 2^−∆∆CT^ method was used for data analysis. Three biological replicates were conducted. The PCR products were sent to a biological company for sequencing to verify the primer specificity. Data analysis and graphing were performed using IBM SPSS Statistics (Version 27.0.1) and GraphPad prism software (Version 10.1.2). The primers used for detection are shown in Table 2.

#### 4.2.7. Transient Transformation of *PmPR1-61* in *P. massoniana* Callus

The *PmPR1*-*61* was cloned and ligated to the pCAMBIA1300-35S-EGFP expression vector through homologous recombination. The Agrobacterium GV3101 was transformed with the empty vector and the constructed pCAMBIA1300-*PmPR1*-*61*-EGFP plasmid, and then cultured in LB medium at 28 °C until the OD_600_ reached 0.6–0.8. After centrifugation, the bacteria were resuspended in a resuspension solution to an OD_600_ of 0.1. The *P. massoniana* callus was immersed in the infection solution and vacuum filtered for 10 min, and the bacterial solution was filtered through a sterile cell sieve. The material was then dried with filter paper and placed on a solid co-culture medium for 48 h in the dark. The callus was collected and immediately frozen in liquid nitrogen and stored at −80 °C.

The content of the plant physiological indicator proline (Pro), and the activities of superoxide dismutase (SOD), catalase (CAT), and polyphenol oxidase (PPO) are classic parameters for evaluating plant disease resistance responses and oxidative damage. The corresponding determination methods (provided by Nanjing Convinced-test Technology Co., Ltd. (Nanjing, China)) were used to measure these indicators. A total of three biological replicates were conducted, and the data were analyzed and graphed using SPSS (Version 27.0.1) and GraphPad prism software (Version 10.1.2).

## 5. Conclusions

This study systematically identified 63 members of the *PmPR1* family in *P. massoniana* and found that this family has undergone a notable expansion in this species while generally exhibiting relatively conserved structural features but markedly diversified expression patterns. Most *PmPR1* genes lacked introns, which may facilitate the rapid activation of defense-related transcription during the early stage of *B. xylophilus* infection. In contrast, differences in conserved motifs, protein properties, and expression patterns suggest that functional diversification accompanied the expansion of this gene family. Several *PmPR1* members formed gene clusters on chromosomes and showed coordinated or specific expression patterns in different resistant materials and at different infection stages, indicating that these gene clusters may play regulatory roles in rapid response and functional specialization. Among them, *PmPR1-55*, *PmPR1-56*, and *PmPR1-61* may serve as important candidate genes involved in the response to pine wood nematode infection, and *PmPR1-61,* in particular, may participate in the early establishment of resistance by regulating ROS homeostasis and enhancing cell wall defense and osmotic protection. This study provides a foundation for elucidating the molecular mechanisms underlying pine wood nematode resistance in *P. massoniana* and for mining candidate resistance genes.

This study still has certain limitations. At present, only the preliminary functional validation of *PmPR1-61* has been conducted, and systematic analyses of H_2_O_2_ accumulation, MDA content, and other related oxidative damage indicators are still lacking. The underlying mechanisms therefore require further verification through stable transformation, protein interaction analyses, and additional physiological and biochemical experiments.

## Figures and Tables

**Table 1:** Basic characteristics, physicochemical properties, and subcellular localization prediction of the identified proteins.

Name	Gene ID	Number of Amino Acid (aa)	Molecular Weight (Da)	Theoretical Isoelectric Point (pI)	Instability Index	Aliphatic Index	GRAVY	Signal Peptide (SP)	Transmembrane Helix (TMH)	Subcellular Localization
PmPR1-1	gmmutg454190G000010.1	169	18,613.01	7.54	45.11	76.75	−0.209	Yes	no	extracellular space
PmPR1-2	gmmutg41797G000030.1	169	18,615.12	8.62	53.06	75.03	−0.186	Yes	no	chloroplast
PmPR1-3	gmmutg15513G000010.1	169	18,818.32	8.1	45.68	73.25	−0.213	Yes	no	chloroplast
PmPR1-4	gmmutg23460G000030.1	261	29,090.14	8.62	41.83	73.6	−0.223	Yes	no	chloroplast
PmPR1-5	gmmutg9222G000010.1	169	18,881.36	6.8	37.85	76.09	−0.223	Yes	no	extracellular space
PmPR1-6	gmmutg9222G000030.1	169	18,936.44	8.47	39.37	73.79	−0.299	Yes	no	chloroplast
PmPR1-7	gmmutg9222G000060.1	169	18,884.37	8.68	43.2	73.2	−0.276	Yes	no	chloroplast
PmPR1-8	gmmutg103154G000010.1	169	18,795.27	8.47	39.25	73.2	−0.256	Yes	no	chloroplast
PmPR1-9	gmmutg42199G000030.1	147	16,399.28	8.2	49.07	57.76	−0.564	no	no	nucleus
PmPR1-10	gmmutg136767G000020.1	169	18,739.16	8.15	44.86	74.44	−0.23	Yes	no	extracellular space
PmPR1-11	gmmutg103154G000020.1	214	24,051.58	8.87	50.23	74.21	−0.104	no	no	chloroplast
PmPR1-12	gmmutg136767G000010.1	161	17,555.69	8.96	49.63	66.71	−0.34	no	no	chloroplast
PmPR1-13	gmmutg23460G000040.1	167	18,733.38	9.46	55.59	71.86	−0.286	Yes	no	chloroplast
PmPR1-14	gmmutg23460G000010.1	167	18,606.23	9.33	49.31	71.86	−0.258	Yes	no	chloroplast
PmPR1-15	gmmutg23460G000020.1	167	18,656.17	9.26	49.75	67.78	−0.283	Yes	no	chloroplast
PmPR1-16	gmmutg41797G000020.1	168	18,728.23	9.12	53.85	69.7	−0.323	Yes	no	chloroplast
PmPR1-17	gmmutg280350G000010.1	168	18,522.05	9.12	49.51	67.38	−0.277	Yes	no	mitochondria
PmPR1-18	gmmutg280350G000020.1	161	17,639.88	8.92	39.18	67.27	−0.297	no	no	chloroplast
PmPR1-19	gmmutg161585G000020.1	148	16,391.62	9.15	51.68	72.5	−0.318	Yes	no	vacuole
PmPR1-20	gmmutg161585G000010.1	161	17,655.92	9.07	43.44	67.89	−0.306	no	no	chloroplast
PmPR1-21	gmmutg44705G000010.1	169	18,735.2	9.02	46.29	77.28	−0.224	Yes	no	chloroplast
PmPR1-22	gmmutg7458G000010.1	169	18,775.27	9.15	46.21	76.15	−0.264	Yes	no	chloroplast
PmPR1-23	gmmutg7458G000020.1	170	18,904.38	8.86	41.15	75.65	−0.189	Yes	no	extracellular space
PmPR1-24	gmmutg7458G000030.1	170	18,897.48	9.01	42.09	80.24	−0.146	Yes	no	extracellular space
PmPR1-25	gmmutg9222G000040.1	185	20,653.17	9.15	46.31	55.35	−0.716	no	no	cytoplasm
PmPR1-26	MSTRG.9754.1.p1	174	19,288.89	8.17	46.7	72.41	−0.16	Yes	no	extracellular space
PmPR1-27	gmmutg2818G000100.1	175	19,576.07	7.63	50	74.23	−0.245	no	no	cytoplasm
PmPR1-28	gmmutg264327G000010.1	154	17,087.15	7.62	46.5	58.38	−0.366	no	no	chloroplast
PmPR1-29	gmmutg22668G000010.1	224	25,581.5	9.16	43.93	72.77	−0.154	Yes	no	extracellular space
PmPR1-30	gmmutg22668G000020.1	177	19,656.42	8.38	42	66.78	−0.134	Yes	no	extracellular space
PmPR1-31	gmmutg153147G000050.1	176	19,643.51	8.75	42.24	66.02	−0.141	Yes	no	extracellular space
PmPR1-32	gmmutg153147G000010.1	176	19,521.35	8.33	43.76	66.02	−0.08	Yes	no	extracellular space
PmPR1-33	gmmutg37108G000020.1	188	21,445.54	9.21	45.44	72.61	−0.344	Yes	no	chloroplast
PmPR1-34	gmmutg71416G000020.1	198	22,530.77	9.3	46.57	72.42	−0.365	Yes	no	chloroplast
PmPR1-35	gmmutg16978G000010.1	178	20,519.16	6.81	45.3	62.47	−0.456	Yes	no	extracellular space
PmPR1-36	MSTRG.33919.1.p1	193	22,262.26	6.41	53.57	67.2	−0.359	Yes	no	extracellular space
PmPR1-37	gmmutg37108G000030.1	180	20,602.49	9.12	43.52	63.44	−0.449	no	no	chloroplast
PmPR1-38	gmmutg1879G000090.1	186	21,194.62	5.1	39.87	59.25	−0.426	Yes	no	extracellular space
PmPR1-39	gmmutg4704G000040.1	177	20,313.88	8.84	44.44	61.19	−0.49	Yes	no	extracellular space
PmPR1-40	gmmutg204539G000010.1	179	19,889.26	5.79	33.75	61.51	−0.517	Yes	no	extracellular space
PmPR1-41	gmmutg43933G000030.1	190	21,275.98	6.22	36.48	64.11	−0.449	Yes	no	extracellular space
PmPR1-42	gmmutg43933G000020.1	186	20,746.33	6.64	33.6	63.39	−0.465	Yes	no	vacuole
PmPR1-43	gmmutg37556G000030.1	176	19,555.05	6.93	37.45	80.34	−0.211	Yes	no	vacuole
PmPR1-44	gmmutg11267G000010.1	170	19,066.28	4.28	42.55	75.71	−0.173	Yes	no	extracellular space
PmPR1-45	gmmutg50621G000010.1	406	45,797.16	8.82	31.26	85.25	−0.272	Yes	no	chloroplast
PmPR1-46	gmmutg50621G000020.1	168	19,091.41	6.87	33.04	69.64	−0.333	Yes	no	chloroplast
PmPR1-47	gmmutg22316G000030.1	169	19,024.29	8.14	36.17	68.11	−0.284	Yes	no	chloroplast
PmPR1-48	gmmutg22316G000060.1	160	18,136.25	7.55	33.14	68.31	−0.342	Yes	no	chloroplast
PmPR1-49	MSTRG.51975.1.p1	162	18,495.58	5.64	43.45	60.19	−0.581	Yes	no	extracellular space
PmPR1-50	gmmutg9810G000080.1	160	18,282.27	6.04	43.42	57.88	−0.664	Yes	no	extracellular space
PmPR1-51	gmmutg20001G000020.1	160	17,993.88	4.84	31.16	64	−0.439	Yes	no	chloroplast
PmPR1-52	gmmutg22316G000020.1	160	17,860.86	4.97	30.51	65.81	−0.369	Yes	no	extracellular space
PmPR1-53	gmmutg71416G000010.1	160	18,000.21	8.38	38.87	63.38	−0.463	Yes	no	chloroplast
PmPR1-54	gmmutg20001G000030.1	167	19,169.18	4.59	49.56	56.05	−0.504	Yes	no	extracellular space
PmPR1-55	gmmutg48626G000010.1	162	17,933.05	7.54	39.07	63.21	−0.394	Yes	no	extracellular space
PmPR1-56	gmmutg17404G000010.1	162	17,730.67	5.83	37.95	62.59	−0.409	Yes	no	chloroplast
PmPR1-57	MSTRG.20082.1.p1	188	20,930.5	5.29	30.22	71.49	−0.262	no	no	vacuole
PmPR1-58	gmmutg73633G000010.1	160	17,603.48	4.6	28.86	67.06	−0.299	Yes	no	chloroplast
PmPR1-59	gmmutg1879G000100.1	131	14,284.02	4.78	28.53	78.93	−0.133	Yes	no	chloroplast
PmPR1-60	gmmutg73633G000030.1	160	17,567.53	4.73	31.42	71.94	−0.247	Yes	no	chloroplast
PmPR1-61	gmmutg4939G000010.1	178	19,797.36	6.88	41.1	73.48	−0.308	no	no	extracellular space
PmPR1-62	gmmutg22750G000010.1	171	18,960.45	5.52	34.69	78.65	−0.116	Yes	no	extracellular space
PmPR1-63	gmmutg12576G000020.1	146	16,424.85	8.7	36.26	93.42	0.027	Yes	no	extracellular space

Note: Name, protein name; Gene ID, gene identifier; Number of amino acids (aa), total number of amino acid residues in the protein; Molecular weight (Da), predicted molecular mass in Daltons; Theoretical isoelectric point (pI), the theoretical pH at which the protein carries no net charge; Instability Index, an estimate of protein stability in vitro; Aliphatic Index, the relative volume occupied by aliphatic side chains; GRAVY, grand average of hydropathicity; Signal Peptide (SP), presence or absence of a signal peptide; Transmembrane Helix (TMH), presence or number of predicted transmembrane helices; Subcellular Localization, predicted cellular compartment of the protein.

**Table 2:** Primers used for qRT-PCR.

Gene	Forward Primer Sequence (5′–3′)	Reverse Primer Sequence (5′–3′)
*PmPR1-45*	TAGGATGTTGCAAGCGAGGG	GATATGCAAGGCCTCGAGCT
*PmPR1-46*	ATCTCCTCACAATGACGCGC	GCTTCCGATGGGGTCTGATA
*PmPR1-48*	GCCAAGATGTGCAGCAACAA	TTAGAGTGCCTCAAAGCGCA
*PmPR1-51*	GTAGGGCAAGGGCAAGATGT	ACATAGTTCCCACGCGGATC
*PmPR1-53*	AGCACTCTGGTGGCCAATAC	CCGCTGTTGCATTGAGCTTT
*PmPR1-55*	GCATTGTACTGCCTAGTCGG	GCGGGTCGTAGTTGCAGATA
*PmPR1-56*	GGCCAAGATGTACAGCAACG	TTTGCCCAACAACGTTTCCC
*PmPR1-57*	ATGTGTTGCATAGTCGGGCA	GCAGTCTCCCACTCGTTGAT
*PmPR1-58*	GACGGGCAAGGGCAAGATAT	TGCCTGGCCGACTACATTTC
*PmPR1-60*	GTCGGGCAAGGGCAAGATAT	TGCCTGGCCGACTACATTTC
*PmPR1-61*	TCAGAGGGATCACGACCACT	ACTCCGCATTGTTGATCCGT
*EF2*	CTGCGATGTCCCTCATGTTA	AACAAGGTCTTTCCCCTCGT

## Data Availability

All the necessary data to evaluate the conclusions in thepaper can be found in the paper itself. Further inquiries can be directed to the corresponding author.

## Abbreviations

The following abbreviations are used in this manuscript:

SOD	Superoxide dismutase
PPO	Polyphenol oxidase
CAT	Catalase
Pro	Proline
